# Therapeutic Options for the Prevention of Thromboses in Cushing’s Syndrome: A Propensity-Matched, Retrospective Cohort Analysis

**DOI:** 10.7759/cureus.84616

**Published:** 2025-05-22

**Authors:** Maxim J Barnett, Sarah Eidbo

**Affiliations:** 1 Internal Medicine, Jefferson Einstein Philadelphia Hospital, Philadelphia, USA

**Keywords:** aspirin, cushing’s disease, endogenous hypercortisolism, low-molecular weight heparin, thromboprophylaxis

## Abstract

Introduction

Cushing’s syndrome, or hypercortisolism, occurs after prolonged exposure to excess cortisol, and can be characterized by moon facies, central fat redistribution, proximal limb muscle weakness and wasting, and abdominal striae. Medical literature points to a relationship between hypercortisolism and hypercoagulability, with higher rates of venous thromboembolism noted. Current guidelines recommend prophylaxis with low-molecular weight heparin (LMWH), but there is little evidence to support LMWH over other forms of anticoagulation.

Methods

We utilized TriNetX US Collaborative Network (TriNetX, LLC, Cambridge, Massachusetts, United States) to investigate the efficacy of different forms of anticoagulation in patients with hypercortisolism, defined by International Classification of Diseases, Tenth Revision (ICD-10) codes. Adult patients with hypercortisolism and prescribed enoxaparin, a form of LMWH, were compared to patients with hypercortisolism prescribed unfractionated heparin, warfarin, apixaban, and aspirin at 81 mg. Groups were propensity-matched according to age at index event, sex, race, ethnicity, and comorbid conditions. The outcomes studied included pulmonary embolism (PE), upper extremity deep vein thrombosis (UE DVT), lower extremity deep venous thrombosis (LE DVT), superficial venous thrombosis (superficial VT), bleeding, transfusion, and all-cause mortality.

Results

No significant differences in outcomes were noted between enoxaparin and heparin, warfarin, or apixaban in patients with hypercortisolism of any cause. Uniquely, the enoxaparin cohort had significantly higher risk of PE, LE DVT, and all-cause mortality compared to the aspirin 81 mg cohort (PE: hazard ratio (HR) 1.697, 95%CI 1.444-1.994, p=0.0345; LE DVT: HR 1.492, 95%CI 1.28-1.738, p=0.0017; mortality: HR 1.272, 95%CI 1.167-1.386, p=0.0002). With further sub-analysis of pituitary-dependent (Cushing’s Disease), enoxaparin continued to demonstrate a higher risk for LE DVT (HR 1.677, 95%CI 1.353-2.079, p=0.0081), and all-cause mortality (HR 1.597, 95%CI 1.422-1.794, p=0.0005).

Conclusion

Although LMWH is currently recommended as the gold standard for anticoagulation in patients with hypercortisolism, our evidence suggests that low-dose antiplatelets such as aspirin 81 mg could outperform it. Further research is warranted to confirm and replicate our findings.

## Introduction

Cortisol is produced within the zona fasciculata of the adrenal cortex and is typically released under stress [1]. Cushing’s Syndrome, first defined in 1912 by American neurosurgeon Harvey Cushing, is a state of prolonged hypercortisolism, presenting with classic phenotypic manifestations, including moon facies, central fat deposition, proximal limb muscle weakness and muscle wasting, and abdominal striae [2]. Cushing’s syndrome can be exogenous (medication-induced/iatrogenic) or endogenous (ectopic adrenocorticotrophic hormone (ACTH), pituitary-dependent, or adrenal adenoma/carcinoma) [3]. Pituitary adenomas causing ACTH-dependent cortisol excess account for 80% of endogenous cases of Cushing's Syndrome and are more specifically termed Cushing's Disease [4]. Overall, however, the most common cause of Cushing’s Syndrome is iatrogenic, from exogenous corticosteroid administration [5].

Hypercortisolism has also been demonstrated to affect coagulation, though the mechanism is unclear [6]. Both venous thromboemboli and pulmonary emboli rates are increased among these patients [7]. The Endocrine Society Guidelines for Treatment of Cushing Syndrome describe altered coagulation profiles that take up to one year to normalize [8]. As a result, limited guidelines recommend prophylactic anticoagulation in Cushing syndrome; while low-molecular-weight heparin (LMWH) is the gold standard, there is little evidence behind this recommendation [9]. Furthermore, few studies assessed individual Cushing’s Syndrome subtypes and associated clotting risks or anticoagulation impact. It is currently unknown whether the antagonistic effects of cortisol will be augmented or hindered by anticoagulation other than LMWH. 

This retrospective multicenter study aimed to address this paucity in data by analyzing differences among various forms of anticoagulation. Patients with Cushing syndrome who were on one of three common anticoagulants, or aspirin, were compared to patients with Cushing's Syndrome on enoxaparin, an LMWH considered the gold standard for prophylaxis in this population. Primary objectives included end-points concerning thromboses (such as pulmonary embolism (PE), upper and lower extremity deep vein thromboses (DVTs), and superficial venous thrombosis (VT)). Secondary objectives included analyzing safety profiles (bleeding, transfusion requirements, and all-cause mortality).

## Materials and methods

Eligibility criteria

**Figure 1:**
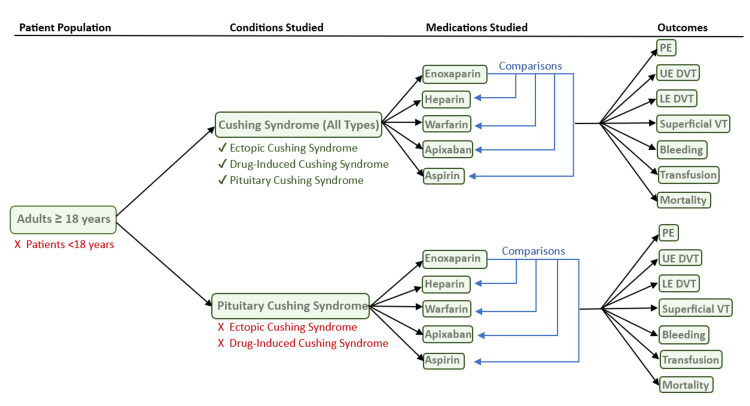
Flow chart for inclusion and exclusion criteria for the study PE: pulmonary embolism; VT: venous thrombosis; DVT: deep vein thrombosis; UE: upper extremity; LE: lower extremity

TriNetX Global Collaborative network (TriNetX, LLC, Cambridge, Massachusetts, United States), a nationwide database of de-identified health data across multiple large healthcare organizations (HCOs), was utilized to compile patients according to International Classification of Diseases, Tenth Revision (ICD-10) codes (Figure 1).

ICD-10 codes included those related to Cushing's Syndrome and one of five studied medications: enoxaparin, heparin, apixaban, warfarin, and aspirin, included in Tables 1 and 2, respectively. ICD-10 codes also included those related to outcomes, including PE, upper extremity (UE) DVT, lower extremity (LE) DVT, superficial VT, bleeding, transfusion, and all-cause mortality (Table 3). Measures of association involved calculating risk differences and relative risks (RRs) with 95% confidence intervals (CIs) to compare the proportion of patients experiencing each outcome across cohorts.

**Table 1:** International Classification of Disease (ICD)-10 codes utilized to identify patients with Cushing Syndrome in the TriNetX database

Cushing’s Syndrome Type	ICD-10 Code
Cushing Syndrome (unspecified)	Drug-Induced Cushing Syndrome (UMLS:ICD10CM:E24.2)
	Other Cushing Syndrome (UMLS:ICD10CM:E24.8)
	Cushing Syndrome, Unspecified (UMLS:ICD10CM:E24.9)
	Pituitary-Dependent Cushing Disease (UMLS:ICD10CM:E24.0)
	Cushing Syndrome (UMLS:ICD10CM:E24)
	Ectopic ACTH Syndrome (UMLS:ICD10CM:E24.3)
Cushing Syndrome (pituitary)	Pituitary-Dependent Cushing Disease (UMLS:ICD10CM:E24.0 )

**Table 2:** International Classification of Disease (ICD)-10 codes utilized to identify anticoagulants and antiplatelets studied in the TriNetX database

Medication	ICD-10 Code
Enoxaparin	NLM:RXNORM:67108
Warfarin	NLM:RXNORM:11289
Heparin	NLM:RXNORM:5224
Apixaban	NLM:RXNORM:1364430
Aspirin	NLM:RXNORM:1191

**Table 3:** International Classification of Disease (ICD)-10 codes utilized to identify outcomes followed in the TriNetX database DVT: Deep Venous Thrombosis; VT: Venous Thrombosis

Outcome		ICD-10 Codes	
Pulmonary Embolism	Pulmonary Embolism		UMLS:ICD10CM:I26
Upper Extremity DVT	Acute embolism and thrombosis of deep veins of unspecified upper extremity		UMLS:ICD10CM:I82.629
	Chronic embolism and thrombosis of deep veins of unspecified upper extremity		UMLS:ICD10CM:I82.729
	Acute embolism and thrombosis of deep veins of right upper extremity		UMLS:ICD10CM:I82.621
	Acute embolism and thrombosis of deep veins of left upper extremity		UMLS:ICD10CM:I82.622
	Acute embolism and thrombosis of deep veins of upper extremity, bilateral		UMLS:ICD10CM:I82.623
	Chronic embolism and thrombosis of deep veins of right upper extremity		UMLS:ICD10CM:I82.721
	Chronic embolism and thrombosis of deep veins of left upper extremity		UMLS:ICD10CM:I82.722
	Chronic embolism and thrombosis of deep veins of upper extremity, bilateral		UMLS:ICD10CM:I82.723
Lower Extremity DVT	Acute embolism and thrombosis of unspecified deep veins of unspecified lower extremity		UMLS:ICD10CM:I82.409
	Chronic embolism and thrombosis of unspecified deep veins of unspecified lower extremity		UMLS:ICD10CM:I82.509
	Chronic embolism and thrombosis of unspecified deep veins of lower extremity		UMLS:ICD10CM:I82.50
	Chronic embolism and thrombosis of unspecified deep veins of lower extremity, bilateral		UMLS:ICD10CM:I82.503
	Acute embolism and thrombosis of unspecified deep veins of lower extremity		UMLS:ICD10CM:I82.40
	Acute embolism and thrombosis of unspecified deep veins of left lower extremity		UMLS:ICD10CM:I82.402
	Acute embolism and thrombosis of unspecified deep veins of right lower extremity		UMLS:ICD10CM:I82.401
	Chronic embolism and thrombosis of unspecified deep veins of left lower extremity		UMLS:ICD10CM:I82.502
	Chronic embolism and thrombosis of unspecified deep veins of right lower extremity		UMLS:ICD10CM:I82.501
	Chronic embolism and thrombosis of left femoral vein		UMLS:ICD10CM:I82.512
	Chronic embolism and thrombosis of right femoral vein		UMLS:ICD10CM:I82.511
	Acute embolism and thrombosis of right iliac vein		UMLS:ICD10CM:I82.421
	Chronic embolism and thrombosis of femoral vein, bilateral		UMLS:ICD10CM:I82.513
	Chronic embolism and thrombosis of unspecified deep veins of unspecified distal lower extremity		UMLS:ICD10CM:I82.5Z9
	Chronic embolism and thrombosis of unspecified tibial vein		UMLS:ICD10CM:I82.549
	Acute embolism and thrombosis of deep veins of lower extremity		UMLS:ICD10CM:I82.4
	Chronic embolism and thrombosis of deep veins of lower extremity		UMLS:ICD10CM:I82.5
	Chronic embolism and thrombosis of other specified deep vein of unspecified lower extremity		UMLS:ICD10CM:I82.599
	Acute embolism and thrombosis of unspecified deep veins of unspecified proximal lower extremity		UMLS:ICD10CM:I82.4Y9
Superficial VT	Embolism and thrombosis of superficial veins of unspecified lower extremity		UMLS:ICD10CM:I82.819
	Acute embolism and thrombosis of superficial veins of unspecified upper extremity		UMLS:ICD10CM:I82.619
	Chronic embolism and thrombosis of superficial veins of unspecified upper extremity		UMLS:ICD10CM:I82.719
Bleeding	Hematemesis		UMLS:ICD10CM:K92.0
	Hemoptysis		UMLS:ICD10CM:R04.2
	Hemorrhage from respiratory passages		UMLS:ICD10CM:R04
	Hemorrhage from other sites in respiratory passages		UMLS:ICD10CM:R04.8
	Hemorrhage from other sites in respiratory passages		UMLS:ICD10CM:R04.89
	Melena		UMLS:ICD10CM:K92.1
	Hemorrhage of anus and rectum		UMLS:ICD10CM:K62.5
	Epistaxis		UMLS:ICD10CM:R04.0
Transfusion	Transfusion of Nonautologous Whole Blood into Peripheral Vein, Percutaneous Approach		UMLS:ICD10PCS:30233H1
	Transfusion of Nonautologous Whole Blood into Central Vein, Percutaneous Approach		UMLS:ICD10PCS:30243H1
	Transfusion of Nonautologous Red Blood Cells into Peripheral Vein, Percutaneous Approach		UMLS:ICD10PCS:30233N1
	Transfusion, blood or blood components		UMLS:CPT:36430
	Transfusion of Nonautologous Red Blood Cells into Central Vein, Percutaneous Approach		UMLS:ICD10PCS:30243N1
	Transfusion of Nonautologous Frozen Red Cells into Peripheral Vein, Percutaneous Approach		UMLS:ICD10PCS:30233P1
	Transfusion of Nonautologous Red Blood Cells into Peripheral Artery, Percutaneous Approach (deprecated 2020)		UMLS:ICD10PCS:30253N1
	Transfusion of Nonautologous Frozen Red Cells into Central Vein, Percutaneous Approach		UMLS:ICD10PCS:30243P1
	Transfusion of Nonautologous Red Blood Cells into Central Artery, Percutaneous Approach (deprecated 2020)		UMLS:ICD10PCS:30263N1
	Transfusion of Nonautologous Frozen Red Cells into Peripheral Artery, Percutaneous Approach (deprecated 2020)		UMLS:ICD10PCS:30253P1
	Transfusion of Nonautologous Frozen Red Cells into Central Artery, Percutaneous Approach (deprecated 2020)		UMLS:ICD10PCS:30263P1
	Transfusion of blood product		UMLS:SNOMED:116859006
	Transfusion of red blood cells		UMLS:SNOMED:116863004
Mortality	Deceased		Deceased (demographic)

Cohort definitions

For each medication listed, two cohorts were compared: (i) a cohort of patients with hypercortisolism on enoxaparin and (ii) a cohort of patients with hypercortisolism on heparin, warfarin, apixaban, or aspirin at 81 mg (Table 4). The cohorts strictly assessed only adult patients (defined as at least 18 years of age); pediatric patients were not analyzed.

**Table 4:** Outputs of healthcare organization queries as defined in corresponding tables HCO: Healthcare Organization

Cohort	Run
Enoxaparin	146 HCOs with 99 providers responding with 12,885 patients
Heparin	145 HCOs with 97 providers responding with 16,376 patients
Warfarin	145 HCOs with 82 providers responding with 3,230 patients
Apixaban	146 HCOs with 91 providers responding with 3,982 patients
Aspirin (81 mg)	144 HCOs with 51 providers responding with 8,200 patients

Statistical analysis

Index events and time windows were defined to analyze patient outcomes. The index event was defined as the first date a patient met the inclusion criteria for a cohort. The time window was defined as the five years after the index event during which a pre-defined outcome could occur. Outcomes of interest were identified using ICD-10 codes as outlined in Table 1, and included PE, UE DVT, LE DVT, superficial VT, bleeding, transfusion, and all-cause mortality. Cohorts were propensity score-matched 1:1 according to age at index event, sex, race and ethnicity, and comorbid conditions, including endocrine, cardiac, pulmonary, gastrointestinal, and genitourinary conditions (Table 5). Propensity score-matching was performed using TriNetX, with a greedy (nearest) neighbor matching algorithm (caliper of 0.1 pooled standard deviations).

**Table 5:** International Classification of Disease (ICD)-10 codes utilized to propensity match cohorts in the TriNetX database

Variable	ICD-10 Code
Demographics	Age at Index (AI)
	Female (F)
	Black/African American (2054-5)
	Male (M)
	White (2106-3)
	American Indian/Alaskan Native (1002-5)
	Unknown Race (UNK)
	Native Hawaiian/Other Pacific Islander (2076-8)
	Unknown Gender (UN)
	Not Hispanic/Latino (2186-5)
	Hispanic/Latino (2135-2)
	Other Race (2131-1)
	Asian (2028-9)
Diagnosis	Endocrine, nutritional and metabolic diseases (E00-E89)
	Factors influencing health status and contact with health services (Z00-Z99)
	Diseases of the musculoskeletal system and connective tissue (M00-M99)
	Diseases of the circulatory system (I00-I99)
	Diseases of the digestive system (K00-K95)
	Diseases of the nervous system (G00-G99)
	Diseases of the respiratory system (J00-J99)
	Diseases of the genitourinary system (N00-N99)
	Diseases of the blood and blood-forming organs and certain disorders involving the immune mechanism (D50-D89)
	Neoplasms (C00-D49)
	Diseases of the skin and subcutaneous tissue (L00-L99)

Three analytical approaches were performed for this study, including measures of association, survival analysis, and frequency analysis. The measure of association analysis involved calculating RRs (and risk differences) with 95%CIs, comparing the proportion of patients across each cohort experiencing an outcome. Survival analysis was performed with Kaplan-Meier estimators (evaluating time-to-event outcomes), with Log-Rank testing incorporated to compare the survival curves. Furthermore, Cox proportional hazard models were incorporated to provide an estimate of the hazard ratios (HR) and 95%CIs. Patients who exited a cohort before the end of the time window were excluded from the survival analysis. The frequency analysis was performed by calculating the proportion of patients in each cohort who experienced an outcome during the defined period of five years.

For statistically significant associations, an E-value was calculated to assess the potential impact of unmeasured confounders, quantifying the minimum strength of association that would be required by an unmeasured confounder to explain the observed effect (beyond our measured covariates); an E-value of above 2.0 was considered modestly robust, and above 3 was considered strongly robust. Additionally, a limited sensitivity analysis assessing Pituitary Cushing's (the most common cause of endogenous Cushing's Syndrome) was performed. All analyses were conducted through TriNetX, with statistical significance defined as a p-value < 0.05.

## Results

Cushing’s syndrome, unspecified

Enoxaparin and Heparin

After propensity-score matching, 8,658 patients were identified in each cohort. The average age at index event for the enoxaparin cohort was 54.5 + 16.5 years, compared to 53.1 + 17.3 years for the heparin cohort. The enoxaparin cohort had 6,216 females (71.8%), compared to 6,000 (69.3%) in the heparin cohort. Within the enoxaparin cohort, 6035 (69.7%) were Caucasian patients, followed by 987 (11.4%) African American patients, 753 (8.7%) Hispanic/Latino patients, and 216 (2.5%) Asian patients. The heparin cohort was similar in ethnicity, with 5,800 (67.0%) Caucasian patients, 1,099 (12.7%) African American patients, 753 (8.7%) Hispanic/Latino patients, and 268 (3.1%) Asian patients. The enoxaparin and heparin cohorts demonstrated no significant differences in PE (HR 1.171, 95%CI 1.017-1.348, p=0.1797), UE DVT (HR 1.067, 95%CI 0.837-1.362, p=0.8051), LE DVT (HR 1.066, 95%CI 0.931-1.222, p=0.1922), superficial VT (HR 0.974, 95%CI 0.672-1.41, p=0.4576), bleeding (HR 0.948, 95%CI 0.855-1.05, p=0.3547), transfusion (HR 0.873, 95%CI 0.786-0.969, p=0.1767), or all-cause mortality (HR 1.036, 95%CI 0.966-1.11, p=0.9954). A comprehensive summary of the results is demonstrated in Table 6.

**Table 6:** Hazard Ratio, 95% Confidence Intervals and p-values for anticoagulation and antiplatelet comparisons in all causes of Cushing's Syndrome HR: hazard ratio; CI: confidence interval; PE: pulmonary embolism; VT: venous thrombosis; DVT: deep vein thrombosis; UE: upper extremity; LE: lower extremity

p-value	Medication 1	Medication 2	PE	UE DVT	LE DVT	S VT	Bleeding	Transfusion	Mortality
	enoxaparin	heparin	0.1797	0.8051	0.1922	0.4576	0.3547	0.1767	0.9954
	enoxaparin	warfarin	0.3828	0.6	0.1963	0.0995	0.7768	0.5715	0.15
	enoxaparin	apixaban	0.6491	0.6275	0.723	0.4198	0.4356	0.4299	0.2628
	enoxaparin	aspirin 81 mg	0.0345	0.587	0.0017	0.4218	0.246	0.2057	0.0002
-	-	-	-	-	-	-	-	-	-
HR	Medication 1	Medication 2	PE	UE DVT	LE DVT	S VT	Bleeding	Transfusion	Mortality
	enoxaparin	heparin	1.171	1.067	1.066	0.974	0.948	0.873	1.036
	enoxaparin	warfarin	0.936	0.969	0.708	0.655	0.961	1.127	1.042
	enoxaparin	apixaban	0.798	0.666	0.684	4.059	0.933	1.089	1.041
	enoxaparin	aspirin 81 mg	1.697	1.398	1.492	1.718	1.107	1.347	1.272
-	-	-	-	-	-	-	-	-	-
95% CIs	Medication 1	Medication 2	PE	UE DVT	LE DVT	Superficial VT	Bleeding	Transfusion	Mortality
	enoxaparin	heparin	1.017-1.348	0.837-1.362	0.931-1.222	0.672-1.41	0.855-1.05	0.786-0.969	0.966-1.11
	enoxaparin	warfarin	0.755-1.161	0.692-1.356	0.583-0.859	0.376-1.142	0.812-1.137	0.95-1.336	0.93-1.167
	enoxaparin	apixaban	0.608-1.047	0.431-1.03	0.593-0.788	1.156-14.258	0.771-1.129	0.892-1.33	0.912-1.189
	enoxaparin	aspirin 81 mg	1.444-1.994	1.06-1.845	1.28-1.738	1.011-2.92	0.986-1.243	1.185-1.532	1.167-1.386

Enoxaparin and Warfarin

After propensity-score matching, 2,786 patients were identified in each cohort. The average age at index event for the enoxaparin cohort was 54.8 + 16.4 years, compared to 58.9 + 15.9 years for the warfarin cohort. The enoxaparin cohort had 2,020 female patients (72.5%) compared to 1,861 (66.8%) in the warfarin cohort. Within the enoxaparin cohort, 2,000 (71.8%) were Caucasian patients, followed by 334 (12.0%) African American patients, 220 (7.98%) Hispanic/Latino patients, and 64 (2.3%) Asian patients. The warfarin cohort was similar, with 2,056 (73.8%) Caucasian patients, 312 (11.2%) African American patients, 170 (6.1%) Hispanic/Latino patients, and 92 (3.3%) Asian patients. The enoxaparin and warfarin cohorts demonstrated no significant differences in PE (HR 0.936, 95%CI 0.755-1.161, p=0.3828), UE DVT (HR 0.969, 95%CI 0.692-1.356, p=0.6), LE DVT (HR 0.708, 95%CI 0.583-0.859, p=0.1963), superficial VT (HR 0.655, 95%CI 0.376-1.142, p=0.0995), bleeding (HR 0.961, 95%CI 0.812-1.137, p=0.7768), transfusion (HR 1.127, 95%CI 0.95-1.336, p=0.5715), or all-cause mortality (HR 1.042, 95%CI 0.93-1.167, p=0.15) (Table 6). 

Enoxaparin and Apixaban

After propensity-score matching, 2,429 patients were identified in each cohort. The average age at index event for the enoxaparin cohort was 54.6 + 16.4 years, compared to 61.2 + 15.2 years for the apixaban cohort. The enoxaparin cohort had 1,746 female patients (71.9%) compared to 1,571 (64.7%) in the apixaban cohort. Within the enoxaparin cohort, 1632 (67.2%) were Caucasian patients, 318 (13.1%) African American patients, 219 (9.0%) Hispanic/Latino patients, and 68 (2.8%) Asian patients. A similar composition was noted in the apixaban cohort, with 1,683 (69.3%) Caucasian patients, 321 (13.2%) African American patients, 141 (5.8%) Hispanic/Latino patients, and 53 (2.2%) Asian patients. The enoxaparin and apixaban cohorts demonstrated no significant differences in PE (HR 0.798, 95%CI 0.608-1.047, p=0.6491), UE DVT (HR 0.666, 95%CI 0.431-1.03, p=0.6275), LE DVT (HR 0.684, 95%CI 0.593-0.788, p=0.723), superficial VT (HR 4.059, 95%CI 1.156-14.258, p=0.4198), bleeding (HR 0.933, 95%CI 0.771-1.129, p=0.4356), transfusion (HR 1.089, 95%CI 0.892-1.33, p=0.4299), or all-cause mortality (HR 1.041, 95%CI 0.912-1.189, p=0.2628) (Table 6). 

Enoxaparin and Aspirin 81 mg

**Figure 2:**
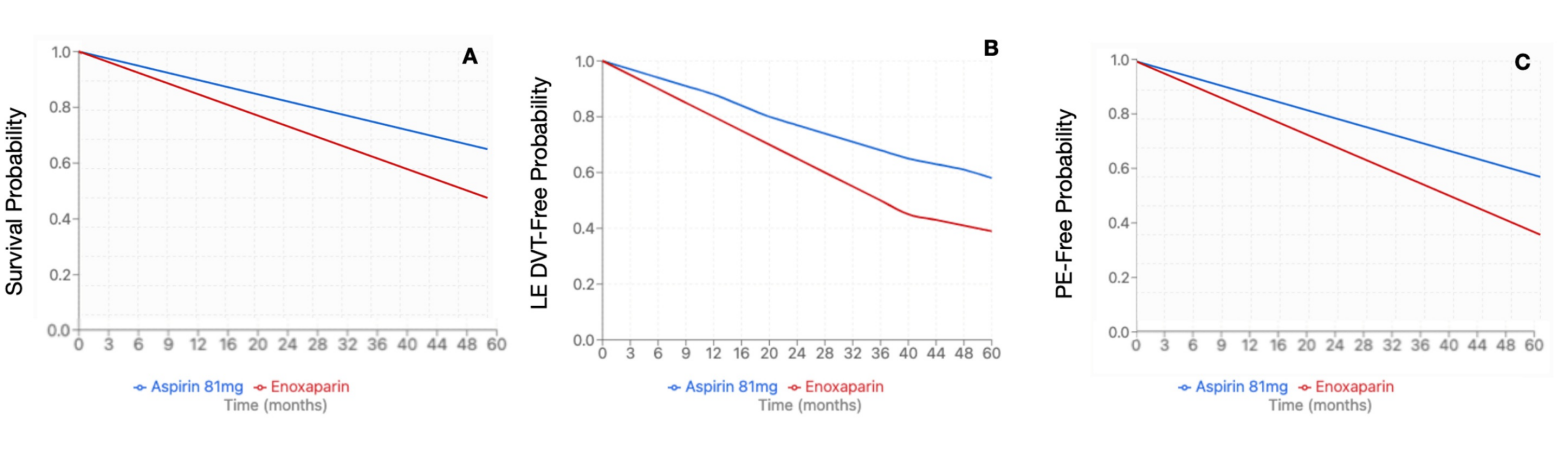
Kaplan-Meier survival curve for pituitary Cushing's subtype (mortality, LE DVT, and PE) (A) Mortality of enoxaparin compared to aspirin 81mg (HR 1.272, 95% CI 1.167-1.386, p=0.0002); (B) LE DVT risk with enoxaparin compared to aspirin 81 mg (HR 1.492, 95%CI 1.28-1.738, p=0.0017); (C) PE risk with enoxaparin compared to aspirin 81 mg (HR: 1.697, 95%CI 1.444-1.994, p=0.0345) DVT: deep vein thrombosis; LE: lower extremity; PE: pulmonary embolism

After propensity-score matching, 6,433 patients were identified in each cohort. The average age at index event for the enoxaparin cohort was 54.5 + 16.6 years, compared to the aspirin 81 mg cohort at 58.8 + 14.9 years. The enoxaparin cohort had 4664 female patients (72.5%) compared to 4,445 (69.1%) in the aspirin 81 mg cohort. Within the enoxaparin cohort, 4,522 (70.3%) were Caucasian patients, followed by 766 (11.9%) African American patients, 521 (8.1%) Hispanic/Latino patients, and 193 (3.0%) Asian patients. Similar demographics were noted within the Aspirin 81 mg cohort, with 4,670 (72.6%) Caucasian patients, 817 (12.7%) African American patients, 425 (6.6%) Hispanic/Latino patients, and 167 (2.6%) Asian patients. The enoxaparin cohort demonstrated a significantly higher risk of PE (HR 1.697, 95%CI 1.444-1.994, p=0.0345), LE DVT (HR 1.492, 95%CI 1.28-1.738, p=0.0017), and all-cause mortality (HR 1.272, 95%CI 1.167-1.386, p=0.0002) compared to the aspirin 81 mg cohort (Figure 2). There was no significant difference in rates of UE DVT (HR 1.398, 95%CI 1.06-1.845, p=0.587), superficial VT (HR 1.718, 95%CI 1.011-2.92, p=0.4268), bleeding (HR 1.107, 95%CI 0.986-1.243, p=0.246), or transfusion (HR 1.347, 95%CI 1.185-1.532, p=0.2057) (Table 6). Due to a significant difference between enoxaparin and Aspirin 81 mg, an E-value was calculated for PE (E-value = 2.783), LE DVT (E-value = 2.348), and all-cause mortality (E-value = 1.860).

Pituitary hypercortisolism (Cushing's disease)

Enoxaparin and Heparin

Propensity-score matching identified 5,602 patients per cohort. The average age at index for the enoxaparin cohort was 53.9 + 16.7 years, compared to 53.7 + 16.9 years in the heparin cohort. The enoxaparin cohort had 4,088 female patients (72.97%) compared to 4,066 (72.58%) in the heparin cohort. The enoxaparin cohort was predominantly Caucasian patients (n=3,948; 70.47%), followed by 641 (11.45%) African American patients, 424 (7.57%) Hispanic/Latino patients, and 139 (2.48%) Asian patients. The heparin cohort was also predominantly Caucasian (n=3,947; 70.46%), followed by 669 (11.94%) African American patients, 401 (7.16%) Hispanic/Latino patients, and 148 (2.64%) Asian patients. There were no significant differences in rates of PE (HR 1.208, 95%CI 1.007 - 1.451, p=0.5803), UE DVT (HR 1.156, 95%CI 0.841 - 1.59, p=0.6863), LE DVT (HR 1.246, 95%CI 1.063 - 1.46, p=0.8996), superficial VT (HR 1.347, 95%CI 0.874 - 2.075, p=0.3731), bleeding (HR 0.916, 95%CI 0.809 - 1.037, p=0.1578), transfusion (HR 0.912, 95%CI 0.798 - 1.042, p=2119), or all-cause mortality (HR 1.02, 95%CI 0.935 - 1.112, p=0.8734). A comprehensive summary of the results is demonstrated in Table 7.

**Table 7:** Hazard ratio, 95% confidence intervals, and p-values for anticoagulation and antiplatelet comparisons in pituitary Cushing's syndrome HR: hazard ratio; CI: confidence interval; PE: pulmonary embolism; VT: venous thrombosis; DVT: deep vein thrombosis; UE: upper extremity; LE: lower extremity

p-value	Medication 1	Medication 2	PE	UE DVT	LE DVT	Superficial VT	Bleeding	Transfusion	Mortality
	enoxaparin	heparin	0.5189	0.2468	0.7586	0.7708	0.5894	0.6273	0.8433
	enoxaparin	warfarin	0.4842	0.7763	0.9651	0.682	0.1996	0.5309	0.399
	enoxaparin	apixaban	0.1047	0.0423	0.647	0.4824	0.2698	0.1122	0.1044
	enoxaparin	aspirin 81 mg	0.9651	0.6358	0.8448	0.9765	0.1167	0.4854	0.5001
-	-	-	-	-	-	-	-	-	-
HR	Medication 1	Medication 2	PE	UE DVT	LE DVT	Superficial VT	Bleeding	Transfusion	Mortality
	enoxaparin	heparin	1.186	1.332	1.232	1.183	0.876	0.963	1.016
	enoxaparin	warfarin	0.804	0.76	0.688	0.815	1.008	1.009	0.976
	enoxaparin	apixaban	0.875	0.761	0.954	3.068	1.084	1.359	1.115
	enoxaparin	aspirin 81 mg	1.173	1.157	1.226	1.165	0.908	0.915	1.028
-	-	-	-	-	-	-	-	-	-
95% CIs	Medication 1	Medication 2	PE	UE DVT	LE DVT	Superficial VT	Bleeding	Transfusion	Mortality
	enoxaparin	heparin	0.983-1.433	0.941-1.885	1.032-1.47	0.776-1.803	0.769-0.998	0.808-1.147	0.929-1.112
	enoxaparin	warfarin	0.612-1.055	0.467-1.235	0.539-0.877	0.447-1.489	0.816-1.246	0.76-1.34	0.843-1.13
	enoxaparin	apixaban	0.659-1.162	0.456-1.271	0.736-1.236	0.843-11.166	0.845-1.381	0.962-1.921	0.944-1.317
	enoxaparin	aspirin 81mg	0.969-1.419	0.827-1.619	1.03-1.46	0.763-1.78	0.797-1.035	0.772-1.085	0.938-1.127

Enoxaparin and Warfarin

Propensity-score matching was performed with 1,694 patients per cohort identified. The average age at index for the enoxaparin cohort was 58.1 + 15.8 years, compared to 58.1 + 15.9 years in the warfarin cohort. The enoxaparin cohort had 1,142 female patients (67.41%) compared to 1,143 (67.47%) in the warfarin cohort. Within the enoxaparin cohort, 1,224 (72.2%) were Caucasian patients, followed by 194 (11.45%) African American patients, 97 (5.73%) Hispanic/Latino patients, and 57 (3.37%) Asian patients. The warfarin cohort had similar demographics, with 1,223 (72.2%) Caucasian patients, followed by 194 (11.45%) African American patients, 102 (6.02%) Hispanic/Latino patients, and 65 (3.84%) Asian patients. There were no significant differences in rates of PE (HR 0.907, 95%CI 0.694 - 1.186, p=0.8117), UE DVT (HR 0.988, 95%CI 0.628 - 1.555, p=0.9848), LE DVT (HR 0.739, 95%CI 0.589 - 0.929, p=0.4445), superficial VT (HR 0.815, 95%CI 0.44 - 1.511, p=0.8098), bleeding (HR 1.001, 95%CI 0.814 - 1.231, p=0.0987), transfusion (HR 1.106, 95%CI 0.889 - 1.376, p=0.4904), or all-cause mortality (HR 0.951, 95%CI 0.83 - 1.089, p=0.1656) (Table 7). 

Enoxaparin and Apixaban

Propensity-score matching identified 1,489 patients per cohort. The enoxaparin cohort was 61.1 + 15.1 years old at the index event, versus the apixaban cohort at 61.4 + 14.9 years. The enoxaparin cohort had 1,054 (70.79%) female patients compared with 1,029 (69.11%) in the apixaban cohort. The enoxaparin cohort was primarily Caucasian patients (n=1,105; 74.21%), followed by 179 (12.02%) African American patients, 74 (4.97%) Hispanic/Latino patients, and 27 (1.81%) Asian patients. The apixaban cohort demonstrated similar demographics with 1,080 (72.53%) Caucasian patients, followed by 180 (12.09%) African American patients, 76 (5.1%) Hispanic/Latino patients, and 27 (1.81%) Asian patients. There were no significant differences in rates of PE (HR 0.949, 95%CI 0.673 - 1.339, p=0.4372), UE DVT (HR 0.832, 95%CI 0.472 - 1.466, p=0.1538), LE DVT (HR 1.166, 95%CI 0.869 - 1.566, p=0.8595), superficial VT (HR 5.323, 95%CI 1.19 - 23.815, p=0.493), bleeding (HR 1.218, 95%CI 0.948 - 1.565, p=0.4021), transfusion (HR 1.319, 95%CI 0.993 - 1.753, p=0.1663), or all-cause mortality (HR 1.131, 95%CI 0.966 - 1.325, p=0.0839) (Table 7). 

Enoxaparin and Aspirin 81 mg

**Figure 3:**
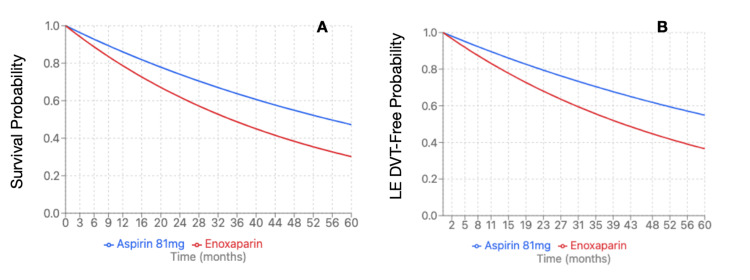
Kaplan-Meier survival curve for pituitary Cushing's subtype (mortality and LE DVT) (A) Mortality of enoxaparin compared to aspirin 81 mg (HR 1.597, 95%CI 1.422-1.794, p=0.0005); (B) LE DVT of enoxaparin compared to aspirin 81 mg (HR 1.677, 95%CI: 1.353-2.079, p=0.0081) HR: hazard ration; DVT: deep vein thrombosis; LE: lower extremity

Propensity-score matching revealed 3,475 patients per cohort. The enoxaparin cohort was 58.8 + 15.3 years at index event, compared to the aspirin cohort at 58.2 + 14.3 years. The enoxaparin cohort had 2,438 (70.16%) female patients compared to the aspirin cohort with 2,445 (70.36%). Within the enoxaparin cohort, 2,539 (73.06%) were Caucasian patients, followed by 378 (10.88%) African American patients, 182 (5.24%) Hispanic/Latino patients, and 74 (2.13%) Asian patients. The aspirin cohort demonstrated similar demographics with 2,554 (73.5%) Caucasian patients, followed by 363 (10.45%) African American patients, 196 (5.64%) Hispanic/Latino patients, and 68 (1.96%) Asian patients. The enoxaparin cohort demonstrated significantly increased risk of LE DVT (HR 1.677, 95%CI 1.353 - 2.079, p=0.0081) and all-cause mortality (HR 1.597, 95%CI 1.422 - 1.794, p=0.0005) (Figure 3). There were no significant differences in rates of PE (HR 1.74, 95%CI 1.354 - 2.236, p=0.2408), UE DVT (HR 1.773, 95%CI 1.108 - 2.837, p=0.8625), superficial VT (HR 4.273, 95%CI 1.969 - 9.273, p=0.5196), bleeding (HR 1.093, 95%CI 0.937 - 1.275, p=0.8554), or transfusion (HR 1.896, 95%CI 1.556 - 2.311, p=0.2609) (Table 7). Due to a significant difference between enoxaparin and Aspirin 81 mg, an E-value was calculated for LE DVT (E-value = 2.744) and all-cause mortality (E-value = 2.574). 

## Discussion

The concept of hypercoagulability in the setting of hypercortisolemia has been documented since the 1970s [10]. Estimates suggest an 18-fold risk of venous thromboembolism in patients with Cushing’s syndrome compared to the general population [11]. Furthermore, venous thromboembolism accounts for up to 11% of all deaths in Cushing’s syndrome [12]. Patients are often noted to have a "coagulation paradox" in Cushing’s syndrome, whereby there is a heightened risk for thrombosis, with concurrent bruising of the skin; thromboembolism is due to an imbalance between pro- and anti-coagulant pathways, whereas bruising is due to atrophy of the skin and capillary fragility [11]. As noted by Feelders and Nieman, two prominent phases for the development of thromboembolic events include the untreated (active) hypercortisolemia and the postoperative phases [11]. Population-based studies have demonstrated a heightened risk for venous thromboembolism prior to diagnosis (in some studies as early as three years before diagnosis) [9].

Despite this heightened risk for venous thromboembolic events, there appears to be a lack of awareness amongst institutions (and individual practitioners), along with improper management. Fleseriu and colleagues, however, do note that in 2020, the awareness of hypercoagulability in Cushing’s syndrome increased around fourfold in two years, with routine prophylaxis increasing to 75% (from 50%) perioperatively (however, most patients only received prophylaxis for up to two weeks postoperatively) [13]. Another survey was performed by the European Reference Network on Rare Endocrine Conditions, noting concerns of heterogeneity with timing, type, and duration of prophylaxis, noting most centers do not have a thromboprophylaxis protocol (identifying only one reference center had a standardized thromboprophylaxis protocol for Cushing’s syndrome) [14]. From the European survey, it was noted that prophylaxis was initiated at diagnosis in 48% of patients, with 17% preoperatively, 26% on the day before (or of) surgery, 13% postoperatively, and 9% "depending on the presentation". With regards to discontinuation of thromboprophylaxis, in centers with a standardized protocol (35% of reference centers), 38% of centers stopped at one month post-operatively, 25% between two and four weeks, and 37% between one week before and two weeks after surgery, between four and six days postoperatively, and at three months postoperatively. When cessation was individualized (in the remaining 65% of reference centers), 60% discontinued thromboprophylaxis once the patient was mobile, 40% with achievement of remission, 27% regarding patient status, and 7% dependent upon hemostatic parameters [14].

There is limited guidance concerning thromboprophylaxis recommendations in Cushing’s syndrome. For example, the Endocrine Society merely recommends assessing the risk of thrombosis in Cushing’s syndrome and administering perioperative prophylaxis if undergoing surgery, but provides no further recommendations [8]. The Pituitary Society highlights the absence of standardized practice for both pre- and postoperative thromboprophylaxis in patients with Cushing’s syndrome [15]. There appears to only be one set of guidelines for thromboprophylaxis in Cushing’s syndrome, known as the “Delphi Panel Consensus”, which forms the basis for the guidelines from the European Society for Endocrinology [9]. The Delphi Panel Consensus recommends considering anticoagulation for all patients with Cushing’s syndrome (in the absence of contraindications), regardless of the underlying etiology, and is recommended in the presence of risk factors [9]. Moreover, thromboprophylaxis is advised to begin at the time of diagnosis [9]. Currently, there is not enough evidence to provide a recommendation for thromboprophylaxis in mild autonomous cortisol secretion [9]. As with any medical patient, thromboprophylaxis should be initiated in all patients with active Cushing’s syndrome who are hospitalized (without contraindications) [9, 15]. Apart from chemical prophylaxis, anti-embolic stockings are not recommended due to the risk of skin fragility and friability [9]. The Delphi Consensus Panel furthermore advises to continue prophylactic anticoagulation for at least three months after biochemical remission (eucortisolemia) has occurred, and note those without additional risk factors (such as obesity, immobility, prior history of venous thromboembolism, or cardiac risk factors) can be considered candidates to stop the medication; one caveat, however, is for patients medically managed with mitotane (which can alter liver function and coagulation factor metabolism), there is an increased risk of bleeding, for which careful monitoring of renal function and bleeding risk is advised [9]. The Pituitary Society provides additional recommendations, such as discontinuing estrogen therapy in women (if used for contraception) [15]. While the Delphi Consensus Panel does not comment upon pediatric patients, the Pituitary Society advises against the use of thromboprophylaxis in the pediatric population due to bleeding risks [15].

**Figure 4:**
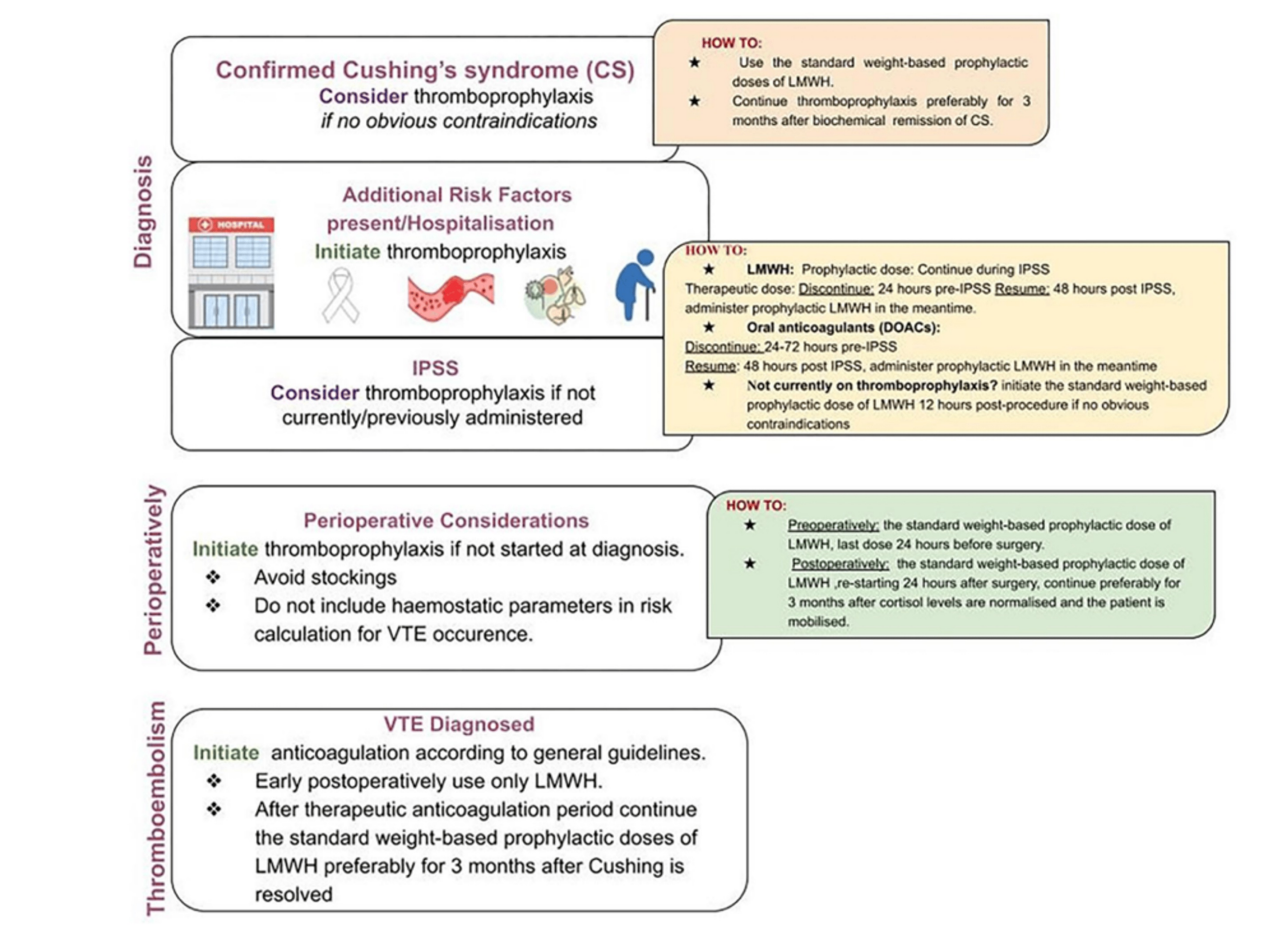
Algorithm for thromboprophylaxis in Cushing's syndrome IPSS: inferior petrosal sinus sampling; VTE: venous thromboembolism; LMWH: low-molecular-weight heparin; DOAC: direct oral anticoagulant Source: Isand et al., 2025 [9]; Published with permission.

The Delphi Consensus Panel furthermore recommend considering thromboprophylaxis at the time of inferior petrosal sinus sampling (if not started before this), due to the risk of thrombosis associated with this intervention; for those who are receiving prophylaxis, it is recommended to continue throughout the procedure, however, if has not been started, it is advised to initiate 12 hours post procedure. Similarly, if thromboprophylaxis was not considered earlier in a patient’s course, it should be reconsidered in the perioperative period, with the last dose of LMWH administered 24 hours prior to surgery and reinitiated 24 hours postoperatively [9]. Isand et al. recommend continuing thromboprophylaxis for three months after cortisol levels normalize (< 5 μg/dL) and when patients can mobilize [9]. In patients for whom a venous thromboembolism develops, patients are advised to receive a therapeutic dose of anticoagulation (preferably LMWH) for three to six months, followed by prophylaxis for three months after resolution of Cushing’s syndrome [9]. The Delphi Consensus Panel provides a summary of their recommendations, shown in Figure 4.

Although intuitively, one may expect the procoagulant profile of Cushing’s syndrome to resolve upon attainment of eucortisolemia with medical management, studies have failed to demonstrate a reduction in venous thromboembolism with medical therapy [16]. Additionally, while one may expect resolution of hypercoagulability with surgical intervention (transsphenoidal sinus surgery or adrenalectomy), the risk maintains in the postoperative period, comparable to that of orthopedic surgery, at times up to one year and beyond to normalize [17]; data from European Register on Cushing’s Syndrome (ERCUSYN) database suggest the risk is greatest six months postoperatively [18]. The estimated risk for postoperative venous thromboembolism in pituitary-dependent Cushing’s is around 4.3% (compared to 0% with a non-functional pituitary adenoma); regarding adrenal surgery, the risk is estimated at around 2.6% [11]. Although the underlying mechanism for the persistent risk for venous thromboembolism remains unknown, it is hypothesized that a sudden drop in cortisol can lead to an inflammatory response (itself activating the coagulation cascade) [16]. Lopes and colleagues note an increase in the number of lymphocytes (because of loss of Th_1_ cell suppression), with increases in cytokines (such as interferon-gamma, interleukin-2, and transforming growth factor-beta) [16]. Comorbidities such as osteoporosis and myopathy (from hypercortisolemia) may be associated with decreased mobility in the postoperative period, influencing the risk for thrombosis [16].

Whilst all subtypes of Cushing’s syndrome can be associated with a heightened risk for venous thromboembolism (pituitary adenoma, adrenal adenoma, medication-induced, ectopic ACTH, and adrenal carcinoma), the latter two are often associated with malignant disease, which itself poses a risk for hypercoagulability from the underlying neoplasm [11]. Patients with Cushing’s syndrome have been found to demonstrate a reduction in activated partial thromboplastin time (aPTT), alongside increases in clot lysis time, procoagulant factors (such as factor VIII, von-Willebrand factor and fibrinogen) and fibrinolysis inhibitors (including plasminogen activator-inhibitor-1, thrombin activatable fibrinolysis inhibitor, and alpha-2 antiplasmin) [11,12,17]. Varlamov et al. have also noted an increase in thrombin, thromboxane A_2_, and platelets. Other studies have additionally demonstrated elevated proteins C and S as well as antithrombin III, which are hypothesized to be increased as a compensatory mechanism from the state of hypercoagulability [12]. Barbot et al. demonstrate elevation in factor VIII and von-Willebrand factor within the first few months after transsphenoidal sinus surgery, along with abnormally large von-Willebrand multimers (which are typically found in the cellular components), which can induce spontaneous platelet aggregation [17].

Lopes et al. note that altered von-Willebrand factor levels are not a constant feature reported in Cushing’s syndrome, and state it depends upon the polymorphism of the gene promoter, providing an example of haplotype 1 of the gene promoter conferring the greatest risk for elevated von-Willebrand factor levels by cortisol [16]. Barbot and colleagues furthermore note ABO blood groupings as an additional influencer of the procoagulant state; as an example, blood group-O patients have a near one-quarter reduction in levels of von-Willebrand factor [17]. Feelders and Nieman note heterogeneity in coagulation profiles based on individual characteristics and differing assay techniques [11]. van Haalen and colleagues note an absence of a correlation between severity of hypercortisolism and hemostatic abnormalities [14]; this is echoed by Varlamov et al., stating there is no linear relationship between coagulation parameters and venous thromboembolic events, nor with urinary free cortisol elevation [12]. Varlamov and colleagues further note that a subset of patients may have unaltered coagulation parameters, for which they advise against stratifying patients’ risk based on coagulation parameters [12]. 

In 2016, Zilio and colleagues posed a scoring system to stratify patients with active Cushing’s syndrome, including both clinical and biochemical parameters, including age (> 69 = 2 points), reduction in mobility (2 points), acute severe infection (1 point), prior cardiovascular event(s) (1 point), midnight plasma cortisol (> 3.15 times upper limit of normal = 1 point), and shortened aPTT (1 point) [19]. Lopes et al. describe the stratification as follows: 2 points (low risk), 3 points (moderate risk), 4 points (high risk), and > 5 points (very high risk) [16]. It should be noted, however, that Zilio et al.'s study was performed on only 176 patients and has not been validated in other studies [19]. Further drawbacks include the failure to account for postoperative events (a major source of venous thromboembolism in Cushing’s syndrome), and despite the stratification categories, no recommendations for treatment are provided.

LMWH is the first-line medication, consistent across differing societies. Despite being the gold standard, there are limited studies demonstrating a beneficial reduction in venous thromboembolic events in such cohorts; similarly, studies are lacking in analysis of the other classes of anticoagulants in head-to-head comparisons against LMWH for thromboprophylaxis in hypercortisolism. Another limitation is the fact that certain studies solely address thromboprophylaxis in the postoperative period. As an example, McCormick et al. performed one of the only trials comparing unfractionated heparin and LMWH (enoxaparin), noting no differences in hemorrhagic complications or thromboses; however, this was analyzed in patients undergoing transsphenoidal sinus surgery [10].

The current study retrospectively analyzed the various anticoagulant agents for the prevention of venous thromboembolism in Cushing’s syndrome (of any subtype), compared to the gold standard, LMWH (in this study, enoxaparin). When analyzing Cushing’s syndrome, our study demonstrated no significant differences in outcomes between enoxaparin and warfarin, apixaban, or unfractionated heparin; however, aspirin 81 mg demonstrated a lower risk of all-cause mortality, PE, and LE DVT. With subanalysis of Cushing’s disease (pituitary-related), there was no significant difference between enoxaparin and warfarin, apixaban or unfractionated heparin; aspirin 81 mg again noted a reduced all-cause mortality and LE DVT (but did not lower the risk of PE, compared with Cushing’s syndrome of all types combined). With E-value sensitivity analysis, the association remained moderately robust with PE (all Cushing's types combined), LE DVT (all Cushing's types and pituitary Cushing's), and mortality (solely pituitary Cushing's), however, mortality was weak-to-moderate with Cushing's syndrome of all types (Table 8).

**Table 8:** E-value sensitivity analyses for significant findings DVT: deep vein thrombosis; LE: lower extremity; PE: pulmonary embolism

Outcome	Hazard Ratio	E-value	Interpretation
PE (All Cushing’s Types)	1.697	2.783	Moderate
LE DVT (All Cushing’s Types)	1.492	2.348	Moderate
LE DVT (Pituitary)	1.677	2.744	Moderate
Mortality (All Cushing’s Types)	1.272	1.860	Weak
Mortality (Pituitary)	1.597	2.574	Moderate

Aspirin, a non-steroidal anti-inflammatory drug, was first identified to irreversibly inhibit platelet function in the 1950s by Dr. Lawrence Craven [20]. Data is scarce in terms of aspirin’s role in thromboprophylaxis in hypercortisolemia. In 1999, Semple and Laws Jr. initially reported the use of aspirin postoperatively for six weeks (starting postoperative day one) in patients with Cushing’s disease who underwent transsphenoidal sinus surgery; while the authors mentioned a reduction in rates of venous thromboemboli, no factual data was provided (including dose of aspirin, complications experienced, and number of venous thromboemboli before and after) [21]. In 2015, Smith et al. performed an additional study with 81 mg of aspirin again administered starting postoperative day one (alongside sequential compression devices and mobilization), reporting that none of the 82 patients developed DVTs (with only two cases of epistaxis) [22]. It was not until 1994, however, in the Antiplatelet Trialists’ Collaborations’ meta-analysis, that aspirin demonstrated a reduced risk for venous thromboembolism, with similar findings replicated in the Pulmonary Embolism Prevention trial in 2000 and the WARFASA (Warfarin and Aspirin) and ASPIRE (Aspirin to prevent recurrent venous thromboembolism) trials in 2012 [23]. In 2012, the American College of Chest Physicians [24,25] were the first to recommend aspirin as thromboprophylaxis following total hip or knee replacement, followed by the National Institute for Health and Care Excellence in 2018 (advising LMWP followed by aspirin) and the American Society of Hematology in 2019 (advising either aspirin or oral anticoagulation after total hip or knee replacement) [25]. Despite recognition of the reduction in venous thromboembolism by aspirin (and its incorporation into guidelines), its role in thromboprophylaxis is largely limited to orthopedic surgery. The mechanisms of aspirin and its reduction in venous thromboembolism is not entirely understood, but believed to occur via differing mechanisms, including inhibition of cyclooxygenase-1 (which reduces thromboxane A_2_, a promoter of platelet aggregation), prevention of thrombin formation and thrombin-mediated coagulant reactions, acetylation of proteins involved in coagulation (such as fibrinogen), and enhancing fibrinolysis [23,26].

Strengths and limitations

To the best of our knowledge, a study specifically comparing the impact of aspirin with that of LMWP in Cushing's syndrome has not been performed; as a result, our study adds to the paucity of literature pertaining to this topic. Notable strengths in the study include a large sample size (allowing robust comparisons amongst treatment arms), incorporation of propensity-score matching (allowing for internal validity through balancing baseline comparison groups), and comprehensive measurable outcomes. 

Limitations to our study are multifold, and include retrospective design, for which intrinsic biases are inherent and can affect causal inference (despite matching techniques). Furthermore, data collection (via TriNetX) relied on correct ICD-10 coding, which could be a source of potential error if conditions and medications are coded improperly, or if our queries missed ICD-10 codes that could also correspond with outcomes. Similarly, TriNetX also relies on queries of healthcare organizations, many of which may not have responded with data, which could inaccurately skew the results. Although TriNetX uses global data, the majority of patient data was derived from the United States population, which could result in less generalizable data to the global public. These findings should be interpreted within the correct context and with caution to prevent misrepresentation. Compliance was a variable that could not be controlled for. Moreover, those who had taken the medication before the index event were excluded from analysis. While aspirin 81 mg demonstrated a reduction in LE DVT and mortality in Cushing’s disease along with PE with Cushing’s syndrome, we only performed a subgroup analysis concerning pituitary-related causes of Cushing’s syndrome (Cushing’s disease); it remains unclear why the risk of PE was not reduced in the latter subgroup. Due to limitations in ICD-10 coding, further subgroup analyses were not performed (such as adrenal adenoma, adrenal adenocarcinoma, or ectopic ACTH syndrome), for which the implications of treating with aspirin 81 mg cannot be inferred from our data. Similarly, further subgroup analyses, such as gender and race, were not performed. Our study assessed adult patients with Cushing's syndrome, and not pediatric patients, which limits the applicability of our findings to such a cohort. Further studies are required to confirm and replicate our findings in a prospective fashion, stratifying subtypes of Cushing’s Syndrome.

## Conclusions

Cushing's syndrome is associated with a heightened risk for venous thromboembolism, regardless of the underlying etiology. Currently, LMWHs such as enoxaparin remain the gold standard for both thromboprophylaxis and treatment in such patients. There is limited data to support superiority over alternative agents. Our study analyzed enoxaparin against warfarin, unfractionated heparin, and apixaban, for which there was no significant risk difference. When compared to aspirin, enoxaparin demonstrated a greater risk for the development of PE, LE DVT, and all-cause mortality. Further prospective trials are required to replicate our findings and confirm the superiority of aspirin over LMWH.
